# Periscope

**Published:** 1888-09

**Authors:** 


### PERISCOPE.

### MEDICINE.

**Optic Atrophy following Chorea.**—One of these very rare cases is recorded in the *Edinburgh Medical Journal*, March, 1888. A youth of seventeen, who had slight chorea when seven years old, and during convalescence suddenly lost sight in the right eye, was admitted at the Royal Infirmary. Ophthalmoscopic examination showed that there was absolute atrophy of the right optic nerve, and both arteries and veins were extremely small. There was no trace of hæmorrhage, and the disc was clearly defined in outline. The left eye was normal in every respect. This condition was attributed to embolism of the central artery; and if such was the case, it lends some support to the embolic theory of the pathology of the disease.

**Sulphurous Acid in Phthisis.**—The resuscitation of an ancient method, used by Galen, of treating this affection seems to have been attended with very encouraging results. The vapour is produced by burning sulphur in a closed chamber, or by means of a lamp containing bisulphide of carbon, or by burning a current of sulphuretted hydrogen gas; and the patient is directed to go on inhaling for as long a period as possible, and to repeat the operation several times daily. Both local and general improvement occurs, and the bacilli rapidly diminish in numbers. Such, at least, is the report given by MM. Delon and Dariex.—*London Medical Recorder*, May, 1888. ARTHUR B. PROWSE, M.D.

**Eye-Troubles in Locomotor Ataxy.**—M. Berger brings forward the results of his experiences on 109 patients:

(1) Diminution of the intra-ocular tension, sometimes on one side only, and coming on in the paralytic stage.

(2) Paralysis of the smooth muscle-fibres of the eyelids, and, as a result, narrowing of the palpebral fissure. Myosis occurs together with this symptom.

(3) Change in the shape of the pupil; it becomes elliptical instead of circular. M. Berger attributes it to paralysis of the vessels of the iris.

The concomitance of these symptoms reminds one of the analogous effects of section of the sympathetic in the neck; but as each of them may occur separately, M. Berger is inclined to attribute them not to actual lesion of this nerve, but to irritation transmitted along it from the spinal cord.—*Le Progrès médical*, June 16th, 1888.

**Preventive Vaccination in Asiatic Cholera.**—M. Pasteur, in the name of M. Gamaleia of Odessa, read a paper on this subject at the meeting of the Academy of Medicine, on August 22nd. M. Gamaleia has applied to cholera two great principles learnt from Pasteur's experiments—that of progressive increase in the intensity of a virus, and that of the use of chemical vaccines. It is easy to produce cholera-vibriones of extreme virulence by passing them through a pigeon after they have been inoculated on a guinea-pig. It then kills the pigeons by causing a "dry" cholera, with exfoliation of the intestinal epithelium; the microbe is to be found in the blood of those pigeons that die.

After some inoculations this microbe acquires so great virulence that one or two drops of the blood of the inoculated pigeons kills fresh pigeons in from eight to twelve hours. This virus also kills guinea-pigs in still smaller doses. It is important to note that all animals of these two kinds succumb to the virulence of the infective process.

This virus, of absolute lethal power, can confer immunity from cholera; a pigeon twice inoculated with an ordinary culture of the cholera-microbe will not be affected by repeated inoculations of the intensified virus. If a cultivation is made of this intensified virus (obtained from the blood of the pigeon through which the virus has been passed) in some nutritive material, and if the culture thus obtained be heated to 120° C. for twenty minutes, in order to completely destroy the contained microbes, it is always found that the heating process has left uninjured in the sterilised culture an active poison. Four c.cc. of this sterilised culture injected into a guinea-pig causes a progressive lowering of temperature, followed by death in from twenty to twenty-four hours. Pigeons also succumb to it with the same morbid phenomena, but only after a dose of 12 c.cc. at a time has been injected. If the injection of this 12 c.cc. is spread over a period of three, four, or five days, the pigeons are not killed, and are now insusceptible of cholera. The most virulent preparation of the virus does not kill them. In the same way guinea-pigs can be protected by injecting the above sterilised culture in doses of 2 c.cc. on two or three occasions. We are now in possession of a protective vaccination for cholera; the method is based on the employment of sterilised vaccines, and possesses all the advantages of chemical vaccinations—that is to say, safety and certainty. These experiments lead us to hope that this method can be applied in the case of human subjects, with the view of protecting them from the ravages of Asiatic cholera.—*Le Progrès médical*, August 25th, 1888.

**Aneurisms and Tubercle-Bacillus.**—In a paper read before the Academy of Medicine, M. Germain Sée stated that he had been struck by the fact, especially since the discovery of the tubercle-bacillus, that patients suffering from aneurism of the aorta often become tubercular, and die of pulmonary phthisis, attended with the formation of cavities, and progressing slowly without fever. This had been observed by Stokes, and numerous instances can be found in the records of Pathological Societies. Out of twenty-four cases of aneurism under his own care, seven were thus affected with pulmonary tuberculosis.

It is now well known that patients with cardiac affections are not exempt from phthisis. M. C. Paul has demonstrated the frequent occurrence of phthisis in cases of narrowing of the pulmonary artery. Aortic aneurism must now be considered one of the agents favouring the development of the tubercle-bacillus. In aneurism, from the disturbance of the circulation there is a stagnation of the venous blood, and diminished activity of the gaseous exchanges between the gases of the blood and those of the atmosphere. In this internal gaseous medium (in the alveoli of the lungs and smallest air-passages) the bacillus appears to multiply when it enters the body by the respiratory tract. In aneurism the air is imperfectly renewed in the whole of the lungs, just as in normal conditions it is imperfectly renewed in the apices as compared with the rest of the lungs. In short, excess of oxygen hinders, deficiency favours, the development of the bacillus. In this way the multiplication of the bacillus is accounted for; the way in which it penetrates into the pulmonary parenchyma in patients of all ages who are affected with aortic aneurism, remains to be shown.

Lately it has been found that ulcerative endocarditis, or even simple endocarditis, with vegetations is caused by a staphilo-coccus; rarely by a strepto-coccus; more rarely still by the tubercle-bacillus. The analogies between endocarditis and endarteritis allow us to suppose the existence of an endarteritis, due to the presence of a microbe. Are the lesions that affect the internal and middle coats of an artery which has undergone aneurismal dilatation caused by a bacillus? If this question is answered in the affirmative, one can easily explain how the bacillus is washed away from its position in the aneurismal wall to the lung, and there multiplies.

In the treatment of aneurism, M. G. Sée relies on iodide of potassium; its mode of action he explains as follows:

(1) It relieves the dyspnœa due to catarrhal affections of the bronchial tubes by liquefying the effused secretions.

(2) It aids the intra-pulmonary circulation, and diminishes venous stagnation.

(3) The volume of the aneurismal tumour is diminished by contraction of the adventitious coat and the tissues surrounding it.

(4) By reducing the size of the tumour, the pressure on the adjacent nerves is relieved, and pain therefore mitigated.

M. G. Sée gives the iodide in the amount of thirty grains daily, divided into three doses, to be taken at the beginning of a meal.

For the relief of pain he has found subcutaneous injections of antipyrin most efficient. When a cardio-vascular tonic is required, he has obtained good results from sulphate of sparteine, and rejects altogether caffeine and digitalis.—*Bulletin de l'Académie de médecine*, August 14th, 1888.

J. MICHELL CLARKE, M.B.

### THERAPEUTICS.

**Primary and Secondary Drug-action.**—In the *Practitioner* for April and May, 1888, Dr. B. Reed discusses this question, and quotes evidence from numerous authorities to show that at least a considerable number of the most prominent sedative drugs have, in certain doses, a primary stimulant action. He says: "I have investigated a large number of others, and find, so far, no exceptions to the induction of Schultz and Peiper, that all paralysing agents primarily stimulate." Again: "The same principles doubtless hold good with regard to the other drugs imperfectly classified according to some of the most conspicuous effects of their physiological doses—as emetics, purgatives, expectorants, diuretics, &c. At all events, so far as my investigations have gone, there is always to be found a relatively small dose which will produce an effect opposite to that of a toxic dose. This is notably true of the purgatives, two of which, rhubarb and castor oil, have long been used for the cure of diarrhœa. These truths, if clearly demonstrated, are highly important, and merit attention. They have been recognised, and either hinted at or enunciated in a more or less complete form, by numerous authors, including many eminent pharmacologists." The practical outcome of a knowledge that medicines possess two opposite actions, either of which may be curative, is that our power of successfully treating disease will be largely increased.

The paper concludes with an illustrative case, in which the above principles were turned to a practical account. A child of thirteen had an attack of typhoid fever, accompanied by profuse diarrhœa, which bismuth, opium, and lead failed to control. As a last resource, podophyllin, in doses of $\frac{1}{120}$th of a grain every third hour, was given. After the third dose improvement commenced, and the diarrhœa was checked completely in twenty-four hours. During convalescence a severe relapse occurred, in which podophyllin now failed. Fowler's solution, however, in doses of $\frac{1}{8}$th of a drop every two hours, succeeded admirably.

[While yet a student I was so much struck, when reading works on Therapeutics, with the evidence of the different action of certain drugs in large and small doses, that I read a paper on "Therapeutic Action" to the St. Mary's Hospital Medical Society, in 1876, in which, after quoting much evidence from various authorities in favour of the existence of this double action, I concluded by suggesting that *all* drugs have the power of causing opposite effects in large and small doses; and, further, that the small doses would be found to have decidedly curative powers in suitable cases, and that thus we should add largely to our present stock of trustworthy remedies. In a case of diarrhœa, accompanied by severe colic and vomiting, due to cold, and which had continued for two days, I ordered a teaspoonful every half-hour of a weak infusion of colocynth pulp. Within four hours, the pain, sickness, and purging had ceased. This certainly appeared to be an instance of *propter hoc;* but if similar experiments were oftener tried, it would be possible soon to arrive at the truth.]

**Pharmacology of Arsenic.**—In the *Canada Medical and Surgical Reporter*, April, 1888, is a paper by Dr. J. Stewart, discussing some of the points in connection with this subject. Discussing the cause of the gastro-intestinal symptoms, he says: "The well-known fact of over-doses of arsenic in bringing about profuse watery stools (closely resembling those seen in Asiatic cholera) is generally explained on the, at first sight, very plausible assumption that, being a powerful irritant, it sets up a severe gastro-enteritis. Now, I believe it is very exceptional for it to bring on this severe inflammatory action. There is no doubt the drug is a powerful escharotic; but it is too slow in its action to account for the sudden and great vomiting and purging which follow its ingestion."

He then gives details of four *post mortems* in cases of arsenic poisoning, in which there was no evidence of inflammation in the stomach and intestines; and says that death was due to great dilatation of the abdominal arterioles, resulting from paralysis of the splanchnic nerves, and causing, secondarily, a sudden and great lowering of blood-pressure in the whole vascular system. The result of this upon the brain causes in rare cases the so-called "arsenicismus cerebro-spinalis," in which deep coma comes on rapidly and proves fatal. The recognition of this cause of death is an all-important factor in the treatment. The pressing indication in such cases is to raise the fallen pressure; for, as in Asiatic cholera, death is brought about by the immense serous loss from the blood. He recommends, therefore, hypodermoclysis of ℥vi. to ℥x. every two or three hours, of a saline solution (common salt, 4; sodium carbonate, 3; and water, 1000).

**Phenacetin** is a white crystalline substance, analogous in composition to antifebrin (acetanilide); inodorous and tasteless; sparingly soluble in water, but more freely in glycerine; insoluble in acid and alkaline liquids, with the exception of glacial acetic acid. With sulphate of copper it gives a green colour, and with perchloride of iron a red. In five-grain doses it reduces febrile temperatures four or five degrees. Except in one instance, where the urine became darkened, no untoward results have been observed. Its action begins to be manifest within half an hour of its ingestion. The patient generally perspires freely and feels drowsy. Sleep often follows, and pain is relieved. Children bear the drug well. Like antipyrin, it has been found useful in neuralgia, rheumatism, and in certain cases of vomiting.—*London Medical Recorder*, April, 1888, and *Practitioner*, May, 1888.

ARTHUR B. PROWSE, M.D.

### OPHTHALMOLOGY.

**Notes from the Seventh International Ophthalmologicial Congress.**—The meeting of the Congress was held in Heidelberg from the 8th till the 11th of August, during a week of splendid weather, under the auspices of the

Ophthalmologische Gesellschaft (the oldest society devoted to this branch of science), which, at the same time, celebrated its twenty-fifth anniversary.

Professor Donders, who has just resigned, at the age of 70 years, the Chair of Physiology at Utrecht, was a most fitting president.

The meetings were held in the Aula of the University, which had been splendidly decorated in the year 1886, on the occasion of the five hundred years' Jubilee. Demonstrations were also given at the Eye Hospital. Some eighty papers were read, and four special discussions were held.

1. "The Cause and Treatment of Strabismus."—The debate on this subject, opened by MM. Landolt (Paris) and Raymond (Turin), contained little beyond the former's communication at the International Congress at Washington last year; and the results of the debate opened by Snell at the British Medical Association meeting held in Dublin. The aim of treatment is to procure binocular vision. All the non-surgical means at our disposal should be exercised before operation is recommended; and the method and extent of the operation must be based upon a careful consideration of the peculiarities present in each and both eyes, even if merely a good permanent cosmetic effect is to be attained. Most of us have experienced the disappointment of seeing a squint outward result some time after a division of the internal rectus, which at first seemed to give a perfect result. The division of a tendon is the destruction of power, which can never be perfectly regained. The more experience I have of muscular advancement, the more confidence I feel in the value of its application.

2. "The Operation of Cataract."—M. Gayet (Lyons) gave a masterly *résumé* of the subject, the most debatable part of which centres in the statements: "Iridectomy is not necessary, it is rarely of use; as far as possible it should not be an incident in the operation (*un incident opératoire*); if it is done, it should be foreseen. . . ." To remove the remains of the cataract, no instrument must be introduced into the anterior chamber; washing out with a gill of water thrown upon the wound, and which enters and forms eddies in the anterior chamber, alternating with massage, suffices for removal of all *débris* and leaves the pupil quite clear. . . ." "A corneal suture may be indicated. . . ." "Incarceration of the iris is the one accident of this method; the others amount to nothing. . . ." "Opacity of the capsule is a frequent result—one always to be dreaded; secondary operations are full of uncertainty and of danger."

Both in England and on the Continent the very large majority of oculists had adopted the method of Von Graefe, viz., peripheral linear incision of the cornea, combined with iridectomy; but a few experienced operators (Augustin Prichard among them) continued the use of the old flap operation, or some modification, by which the iris is left intact, and the great advantages of a movable pupil are retained. During the late year or two a very marked reaction has taken place in favour of operating without iridectomy. The new school relies much on the prevention of prolapse by use of myotics, and on the improved results under strict antisepsis in the prevention of inflammation of the wound and of the iris, although the latter may have been over-freely stretched at the time of the delivery of the lens, and even bruised in its replacement through the corneal wound into the anterior chamber. Under the liberal use of eserin, the pupil contracts and helps to prevent the after prolapse of the iris through the wound. Despite all precautions, however, the tendency to prolapse is very great, and incarceration had certainly taken place in most of the cases that I had an opportunity of seeing in the clinic of one of the leading advocates of this method.

None would deny the advantage of retaining the perfect pupil, but in endeavouring to do so the risks of prolapse are enormous; whereby the sound pupil is spoiled, healing is seriously prolonged, and the resulting anterior synechia is an ever-present source of danger.

The results communicated to the Congress by Knapp (the first hundred cases are reported in the *Arch. of Ophthalm.*, Vol. xvii., No. 1, 1888) show that he has brought extraction without iridectomy to great perfection; and Schweigger is also a warm advocate of the method. Prof. Hirschberg, whom I thought a year ago to be opposed to it, tells me that he also is now in its favour. In the face of the opinion of these authorities, and of Sattler and others scarcely less weighty, I should expect the question pertinently asked by Anderson Critchett, in his remarks in the debate ("Which would any of us, himself the subject of cataract, select as his friend to extract the lens? One well versed in the method without iridectomy, or one who would perform iridectomy as an essential part of the operation?"), to be answered by a large majority in favour of the latter procedure. There is another important point: Knapp, in his cases, used very freely and frequently a secondary operation for opaque capsule; this, Gayet urges, is a dangerous operation; and in a conversation I had with Snellen, he spoke very strongly of his dread of the apparently slight operation of discission. I think he would have argued that opaque capsule is more likely to follow extraction without iridectomy, than where iridectomy is done; and that, therefore, it is safer to do iridectomy, in that the necessity for a secondary operation is thereby lessened. Knapp, on the other hand, although he lays stress upon the dangers of careless interference, especially in cases of pseudo-membranes following iritis or irido-cyclitis, is a strong advocate of further interference, so as to give the best possible acuteness of vision. My own feeling is that discission or some form of capsulotomy is safer and more easily made available where a coloboma exists. The time is rapidly approaching when the advantages and disadvantages of the iridectomy as a part of the operation for extraction of cataract will be warmly contested, and the report of the proceedings of our Congress will contain weighty arguments in favour of a return to the flap operation.

A story is told that a well-known English oculist said to Knapp, "So you have not yet given up extraction without iridectomy?" To whom the latter said, "And you have not yet commenced to do it."

3. Priestley Smith's communication on "Glaucoma," to the elucidation of which he has devoted so much thought and mechanical intelligence, was most enthusiastically received. I append his abstract on the **Pathology** of the disease:—

"Glaucoma consists in an excess of pressure within the eye, *plus* the causes and the consequences of that excess. Pressure is an essential factor. (A glaucoma without increased tension is probably a glaucoma examined during the intermissions of increased tension).

The pressure of the intra-ocular fluids is determined by three conditions:

*a*. The condition of the secreting organs.

*b*. The condition of the outlets.

*c*. The condition of the fluids themselves.

The aqueous and vitreous fluids are secreted by the ciliary portion of the uveal tract. The aqueous escapes at the angle of the anterior chamber (filtration angle). The vitreous fluid escapes at the papilla, very slowly as compared with the aqueous. Any surplus fluid in the vitreous can pass easily, in the healthy eye, into the aqueous chamber. The condition of the papilla cannot have much influence on the intra-ocular pressure. An albuminous fluid escapes from the anterior chamber much less rapidly than a normal salt-solution under the same pressure.

The chief factors which can raise the intra-ocular pressure are therefore: *a*, hypersecretion by the ciliary processes; *b*, obstruction of the filtration angle; *c*, serosity of the fluids.

*a*. Hypersecretion is sometimes the exciting cause of the attack, but the glaucoma-process cannot be explained by the hypothesis of a persistent hypersecretion.

*b*. Obstruction at the filtration angle is present in most cases of glaucoma; the angle is compressed or closed. Experiment proves that, when the iris-base is pushed forwards, filtration is greatly retarded. It is true that the filtration angle is sometimes closed in eyes which have no glaucoma, but in such eyes there are other changes which render glaucoma an impossibility; the fluid escapes by abnormal outlets, or it is no longer secreted.

*c*. Serosity of the fluids is present in many forms of glaucoma. It is a very important factor in the secondary glaucoma of serous iritis and kerato-iritis; in these cases the filtration angle is widely open and the chamber deep.

**In most forms of glaucoma the filtration angle is closed.** What are the antecedent changes? When they are invisible, we call the glaucoma 'primary'; when they are visible, we call it 'secondary.'

In certain forms of *secondary glaucoma* we can see the manner in which the filtration angle is closed. In every form, except those in which the aqueous chamber is distended by serous fluid, the iris-base is found on dissection to be pushed forwards against the cornea.

In *primary glaucoma* of recent date, dissection shows the iris-base pushed forwards by the swollen ciliary processes, and in many cases the processes are themselves pushed forwards by the lens and zonula.

*a*. The chief predisposing cause is an insufficient circumlental space. Thus the liability to glaucoma increases with age, because the lens grows larger as life advances. Again, the liability is greater in the hyperopic eye, because the ciliary muscle and processes are more prominent in the direction of the lens. Again, a small cornea appears to predispose to primary glaucoma: in 227 persons measured with a special keratometer the average horizontal diameter of the cornea was 11.52 mm; in 52 persons suffering from primary glaucoma in one or both eyes it was 11.02 mm. A cornea measuring 10.5 mm., or less, is exceptional; among the unaffected persons it was found in about four per cent., among affected persons in 31 per cent. It is at present uncertain whether the small cornea of such eyes is a congenital peculiarity or a senile change. (The investigation is not yet completed.)

Senile changes in the vitreous which obstruct filtration into the aqueous chamber are perhaps among the predisposing causes. Perhaps, also, in minor degree, senile rigidity of the sclera and senile degeneration of the blood-vessels.

*b*. The chief exciting causes are those conditions which overfill the uveal tract with blood. General disturbances which depress the circulation and overfill the venous system are the usual antecedents. The ciliary processes swell and, by reason of the insufficient circumlental space, push forward the iris-base and compress the filtration angle. Obstructive phlebitis would do this, but there is no evidence that this is a common antecedent of glaucoma.

Atropine, under predisposing conditions, excites glaucoma by thickening the iris-base.

Glaucoma aggravates itself, because increasing pressure on the choroidal veins causes increasing congestion of the ciliary processes and increasing compression of the filtration angle.

The anatomical predisposition and the vascular disturbance are complementary to each other in varying proportions. Acute glaucoma presents the maximum, chronic non-congestive glaucoma, the minimum of vascular disturbance."

Snellen, from his large clinical experience and operative ability, was well chosen to introduce the question of the **Treatment** of Glaucoma, in which he advocated a new departure, or at least a further development and modification of De Wecker's views. He insists on the necessity for continuous use of myotics, and believes that sclerotomy is far preferable to iridectomy, by leaving the contractile action of the pupil unimpaired. He simply makes a sclero-corneal incision with a Jaeger knife. The suggestion is especially interesting if taken in connection with the recent views against iridectomy in the treatment of cataract already alluded to. He says:

"From the clinical standpoint, glaucoma posterius (relative overfulness of vitreous chamber) must be strictly distinguished from glaucoma anterius (relative overfulness of anterior chamber: iritis serosa, keratitis diffusa).

In *glaucoma posterius* myotics tend to reopen Fontana's spaces by stretching the iris and contracting the meridional fibres of ciliary muscle. They excite the circulation.

In *glaucoma anterius* myotics are prejudicial by extending the surface of the iris and by provoking pupillary adhesions.

Mydriatics act in the opposite way.

Sclerotomy is indicated in all cases of increased tension (hypertonus); it benefits by evacuating serous fluids, loosening peripheral or pupillary iris-adhesions and readmitting the impeded circulation.

The direct thrust of the iridectomy-knive involves less danger of prolapse of the iris than the cut from within outwards of the cataract-knife.

Myotics are a *sine quâ non* in performing sclerotomy. The myotic contraction of the iris prevents prolapse; the contraction of the uveal tract *in toto* promotes the outflow of the fluids, and diminishes the pressure of the choroid against the sclera.

The myotic contraction of the meridional fibres of the ciliary muscle stretches Descemet's membrane, distends the inner mouth of the sclerotomy-wound, and promotes the formation of new channels to Schlemm's canal.

Excision of the iris is a subordinate part of the glaucoma-operation; but it is indicated when the iris tends to prolapse, and when the aqueous humour is retained behind the iris.

The divided sphincter of the iris stretches the iris-periphery less effectually than the undivided sphincter. Sclerotomy without iridectomy permits repetition of operative treatment.

Impending hypertonus should interdict all straining of the accommodation. Schoen's theory is a valuable attempt to find the primary cause of hypertonus in the function of the eye.

Although the details of Schoen's explanation seem objectionable, it may prove true that straining of a diminished accommodation is a cause of glaucoma.

When the elasticity of the lens is lost, contraction of the circular fibres of the ciliary muscle would relax the suspensory ligament, and this relaxation would tend to a forward movement of the lens and ciliary processes.

In glaucoma perfectum, extirpation is indicated by impending pain, and because of its occasional association with intra-ocular tumour.

Extirpation is preferable to exenteration; among other reasons, in the interests of pathological examination."

4. Leber's (Gottingen) paper on "Signification of Bacteriology in Ophthalmology" comprises years of work by one who is, perhaps, the greatest living authority on Ophthalmic Surgery; and, together with Sattler's (Prague) communication, should be studied in full, not only by specialists, but by all who are interested in the association of septic agencies with disease.

Space only admits of our thus briefly noting the four main subjects of discussion. A complete account of the proceedings of the Congress will be published in due time.

The town of Heidelberg was *en fête* and gaily decorated in honour of the Congress.

Some two hundred and fifty oculists registered their names. About twenty were Americans, and eleven represented this country: Critchett, Nettleship, Gunn, and Jessop (London); Argyll Robertson, and Berry (Edinburgh); Swanzy (Dublin); Little and Hill Griffith (Manchester); Priestley Smith (Birmingham).

The social side of the Congress was well maintained; Professor Becker was profuse in his hospitality. The arrangements included evening receptions in the "Schlossrestauration" and the "Stadtgarten." The dinner was held in the Sanatorium, near the Schloss, and was attended by the Burgomeister, the Rector of the University, and many ladies. The usual speeches were made between the various courses.

The Congress was closed by a boat excursion on the Neckar, from Ziegelhausen, together with a grand illumination of the Old Castle, and fireworks, arranged by the city of Heidelberg.

The executive of the Congress were Otto Becker, Hess (Mainz), Stilling (Strasburg), and Bernheimer; and they are to be congratulated on their successful efforts.

The city of Edinburgh has been chosen for the next International gathering of Ophthalmic Surgeons in 1894.

F. Richardson Cross.

